# Elastic Anomaly and Polyamorphic Transition in (La, Ce)-based Bulk Metallic Glass under Pressure

**DOI:** 10.1038/s41598-017-00737-0

**Published:** 2017-04-07

**Authors:** Xintong Qi, Yongtao Zou, Xuebing Wang, Ting Chen, David O. Welch, Jianzhong Jiang, Baosheng Li

**Affiliations:** 1 Department of Geosciences, Stony Brook University, Stony Brook, N.Y. 11794 USA; 2 State Key Laboratory of Superhard Materials, College of Physics, Jilin University, Changchun, 130012 P.R. China; 3 Condensed Matter Physics and Materials Science Department, Brookhaven National Laboratory, Upton, N.Y. 11973 USA; 4 International Center for New-Structured Materials (ICNSM), Laboratory of New-Structured Materials, State Key laboratory of Silicon Materials, and School of Materials Science and Engineering, Zhejiang University, Hangzhou, 310027 P.R. China; 5 Mineral Physics Institute, Stony Brook University, Stony Brook, N.Y. 11794 USA

## Abstract

Pressure-induced polyamorphism in Ce-based metallic glass has attracted significant interest in condensed matter physics. In this paper, we discover that in association with the polyamorphism of La_32_Ce_32_Al_16_Ni_5_Cu_15_ bulk metallic glass, the acoustic velocities, measured up to 12.3 GPa using ultrasonic interferometry, exhibit velocity minima at 1.8 GPa for P wave and 3.2 GPa for S wave. The low and high density amorphous states are distinguished by their distinct pressure derivatives of the bulk and shear moduli. The elasticity, permanent densification, and polyamorphic transition are interpreted by the topological rearrangement of solute-centered clusters in medium-range order (MRO) mediated by the 4*f* electron delocalization of Ce under pressure. The precisely measured acoustic wave travel times which were used to derive the velocities and densities provided unprecedented data to document the evolution of the bulk and shear elastic moduli associated with a polyamorphic transition in La_32_Ce_32_Al_16_Ni_5_Cu_15_ bulk metallic glass and can shed new light on the mechanisms of polyamorphism and structural evolution in metallic glasses under pressure.

## Introduction

Amorphous-to-amorphous phase transitions, also known as polyamorphism, play an important role in the fundamental study of amorphous materials. Pressure-induced polyamorphic transitions have been reported in various materials, such as amorphous ice, vitreous SiO_2_, and also in elemental solids such as silicon, germanium and selenium^1–8^. Recently, metallic glasses (MGs) with non-directional bonding and densely-packed structure have been realized to have the potential to undergo phase transitions under pressure^9–16^. Ce-based MGs are the most extensively studied MGs that have been proved to exhibit polyamorphism under pressure. Combining *in situ* x-ray diffraction observation and *ab initio* calculation, two amorphous polymorphs in Ce_55_Al_45_ metallic glass were reported by Sheng *et al*., and the transition from the low-density amorphous (LDA) state to the high-density amorphous (HDA) state between 2–13.5 GPa was found to be sluggish and hysteretic^11^. Similar results were found in Ce_75_Al_25_ metallic glass by Zeng *et al*. using high-pressure synchrotron x-ray diffraction and x-ray absorption probes, and the transition was reported to occur within the pressure range of 1.5–5 GPa, with a large volume collapse of about 8.6% which coincides with the volume collapse associated with the isostructural transition from α-Ce to γ-Ce transition^12^. More recently, Duarte *et al*. reported the observation of three different amorphous phases for the Ce_70_Al_10_Ni_10_Cu_10_ bulk metallic glass (BMG) in the 0–25 GPa pressure range using inelastic x-ray scattering (IXS) coupled with high-resolution XRD^13^. Their experimental results indicated an initial decrease of longitudinal velocity for pressure up to 0.4 GPa, followed by a substantially higher velocity at ~5 GPa, but with no measurements within the range 0.4–5 GPa as the pressure-induced polyamorphic transition was occurring. It has been postulated that these pressure-induced polyamorphic transitions in lanthanide-based metallic glasses may originate from the delocalization of 4*f*-electrons with pressure^11, 12^; recently, experimental evidences from Ce-L_3_ edge X-ray absorption studies confirm that progressive delocalization of 4*f* electrons under pressure indeed occurs in Ce_75_Al_25_^12^ and Ce_60_Al_20_Cu_20_^14^. Nevertheless, the manifestation of the 4*f*-electron driven amorphous-to-amorphous transition in the bulk elastic properties associated with the large volume collapse and local atomic structure change are still obscure at present, especially for the shear wave velocity and shear modulus. Unlike typical network-forming glasses with open local environment, BMGs are densely packed and disordered. Despite lacking long-range order, the atomic scale short-range order (SRO) and nanoscale medium-range order (MRO) are sufficiently pronounced to dominate their internal structures^17–20^. Thus, measurements of the bulk properties under compression, such as the bulk and shear elastic moduli and specific density, are of critical importance in understanding and evaluating previous structural models that have been derived based on the responses of SRO and/or MRO to external pressures.

Pressure-induced polyamorphism in MGs is reportedly accompanied by a continuous change in density which is difficult to identify by x-ray diffraction method due to the lack of Bragg peaks. The first sharp diffraction peak (FSDP), a ubiquitous feature in the x-ray diffraction patterns of amorphous materials, has been most widely used in probing structural variations of metallic glasses up to intermediate-range order (IRO) scale, as well as to estimate the bulk density under pressure in analogous to the use of Bragg peaks in their crystalline counterparts. However, it has been demonstrated recently by both experimental and theoretical studies that MRO in BMGs displays fractal network characteristics, thus the atomic volume correlates with the position of the FSDP (*q*_*1*_) by a power-law relationship with a fractal dimensionality of 2.3–2.5^20–22^, as compared to the cubic power relationship in crystalline solids. Here we investigate the pressure-induced polyamorphic transition in La_32_Ce_32_Al_16_Ni_5_Cu_15_ MG by measuring its compressional and shear velocities using a state-of-the-art ultrasonic interferometry technique. As demonstrated in a previous study of GeSe_2_ glass under pressure^23^, ultrasonic measurement of acoustic velocities is a unique and powerful tool to detect phase transitions, especially for second-order phase transitions in non-crystalline materials. Moreover, the mass density under pressure can be determined precisely using an iterative procedure based on the measured acoustic velocities^24^. Data from these unique techniques, together with previous data from X-ray diffraction, shed new light on the mechanism of polyamorphism in MGs from the perspective of structural ordering at various length scales.

## Results

**Figure 1:**
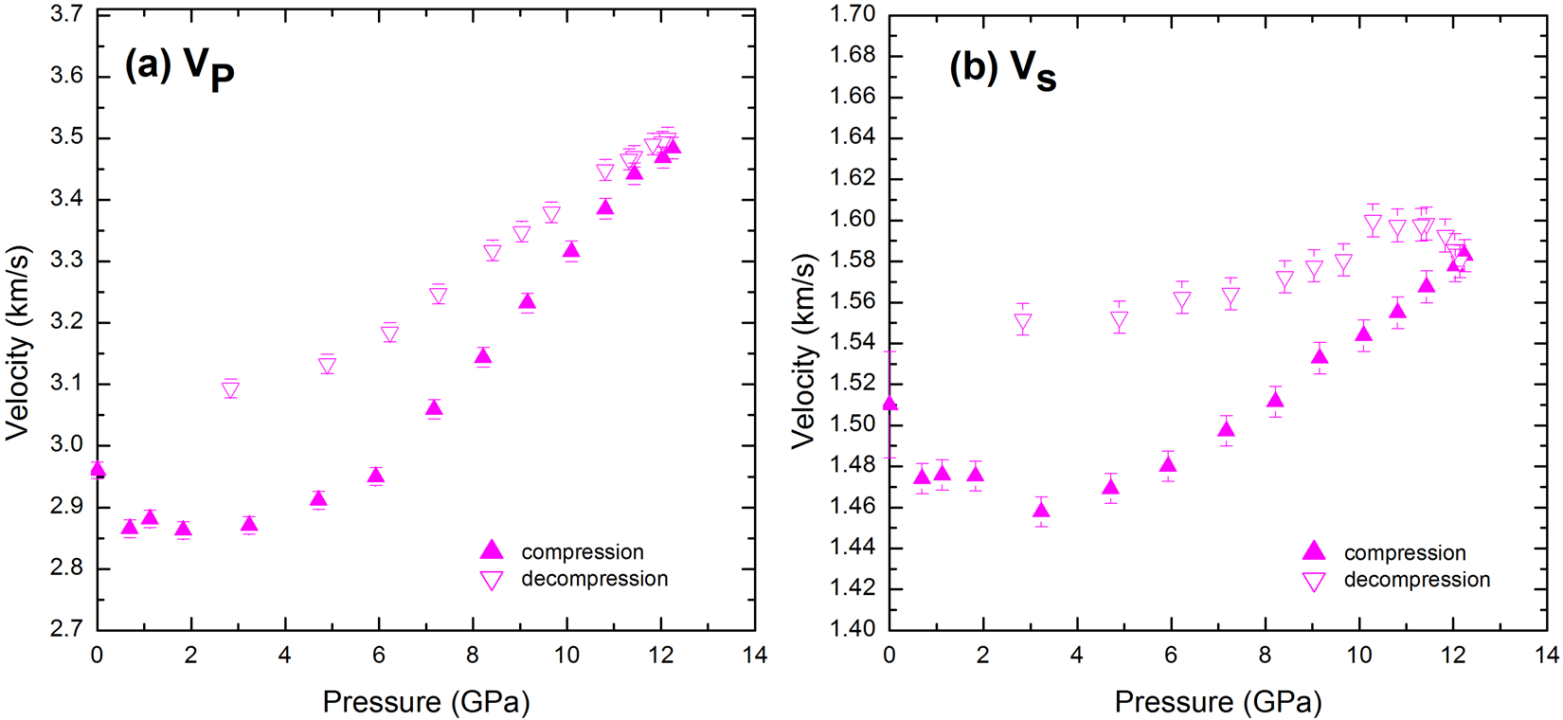
The elastic wave velocities of the La_32_Ce_32_Al_16_Ni_5_Cu_15_ MG as a function of pressure at ambient temperature; (**a**) P wave, (**b**) S wave. Solid symbols are data on pressurization and open symbols are data on depressurization.

The compressional (*P*) and shear (*S*) wave velocities (ν_P_ and ν_S_) of La_32_Ce_32_Al_16_Ni_5_Cu_15_ MG were measured simultaneously under quasi-hydrostatic high pressures up to 12.3 GPa at room temperature in a multi-anvil apparatus. Both *P* and *S* wave velocities exhibit anomalous behavior at pressures between 0–5 GPa during compression (Fig. 1). The velocities upon decompression did not retrace the behavior during compression, which is consistent with the hysteretic densification observed in other Ce-based MGs^11^. Compared to the values at ambient conditions before pressurization (2.96 km/s and 1.51 km/s for ν_P_ and ν_S_, respectively), a pressure-induced decrease of ~3.0% in ν_P_ and ~2.6% in ν_S_ is observed at 0.7 GPa. Such anomalous elastic softening behavior has also been observed in Ce_70_Al_10_Ni_10_Cu_10_ metallic glass by Zhang *et al*.^25^ and Duarte *et al*.^13^ who reported a negative pressure dependence of acoustic velocities up to ~0.5 GPa. At pressures between 0.4 and 5 GPa, a linear rise in the compressional wave velocity is postulated due to the lack of direct measurement within this pressures range^13^. By contrast, our results clearly demonstrate that, after initially decreasing to 0.5 GPa, the compressional and shear velocities continue to display a weak pressure dependence up to 3.2 GPa, beyond which the velocities increase monotonically with pressure. This elastic softening behavior of ν_P_ and ν_S_ under pressure is very similar to the characteristic features displayed by many network glasses undergoing structural modifications during densification, such as amorphous SiO_2_, GeSe_2_ and MgSiO_3_, as detected by Brillouin scattering and/or ultrasonic methods^7, 23, 26–29^, but has rarely been studied in MGs. The prominent changes in the slopes of the pressure dependence for both P and S velocities suggest that the current MG sample La_32_Ce_32_Al_16_Ni_5_Cu_15_ may undergo similar structural modifications towards a densified state in the applied pressure region. For instance, the softening of these long wavelength acoustic phonons has prompted Wang *et al*. to suggest that there exists a certain degree of directional bonding among the constituent atoms in the LDA state of Ce-based MGs^30^, although direct evidence to support this hypothesis is still lacking.

On decompression, neither ν_P_ nor ν_S_ follows its respective path along compression, and both exhibit higher values than those obtained at the same pressure during compression. This suggests that the densification and relaxation follow different pathways in terms of structural modification, presumably due to the occurrence of permanent densification. In addition, a noticeable increase in *S* wave velocity (~0.25 km/s, Fig. 1b) was observed at the beginning of decompression from 12.3 GPa to ~11 GPa (~8 hours). During this period, the S wave travel times of the sample exhibited positive pressure dependence which may be interpreted as a sign of time-dependent densification as observed previously in silicate glass^31^.

**Figure 2:**
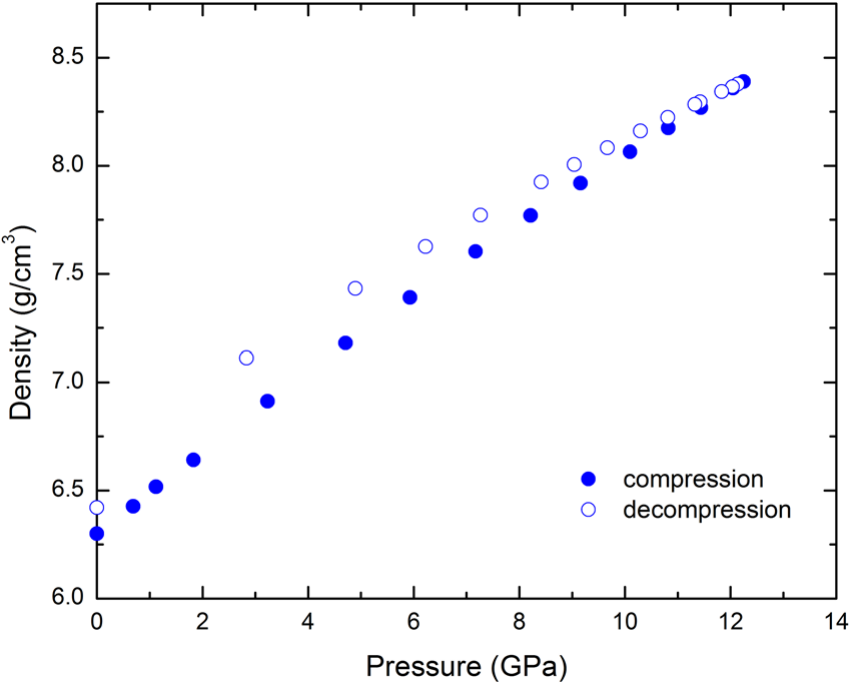
Densities of the La_32_Ce_32_Al_16_Ni_5_Cu_15_ BMG as a function of pressure at room temperature. Solid symbols are data on pressurization and open symbols are data on depressurization. Error bars are about the size of the symbols.

The mass densities at high pressures are derived using the measured *P* and *S* wave velocities and the initial density at ambient conditions (see Method), and the results are shown in Fig. 2. It is interesting to note that the resultant density exhibits a gradual and smooth increase during compression in spite of the anomalous changes in velocities over the current pressure range (0–12.3 GPa). The 1.90% increase in the density for the current La_32_Ce_32_Al_16_Ni_5_Cu_15_ sample at pressure about 0.69 GPa can be compared with the values from the previous acoustic study on Ce_70_Al_10_Ni_10_Cu_10_ MG, in which a 1.89% density increase was reported at its peak pressure of 0.5 GPa^25^. At the peak pressure of (~12.3 GPa) the current study, the density of La_32_Ce_32_Al_16_Ni_5_Cu_15_ reaches ~8.26 g cm^−3^, yielding a more dramatic increase (~31.1%) compared to that of Zr-based MG (e.g., Zr_46_Cu_37.6_Ag_8.4_, ~10%)^32^ as well as of other MGs (~6–10%)^11, 33^. Upon decompression, the densities show hysteresis in comparison with those along compression, which leads to a permanent residual densification about 1.9% upon recovery at ambient conditions.

**Figure 3:**
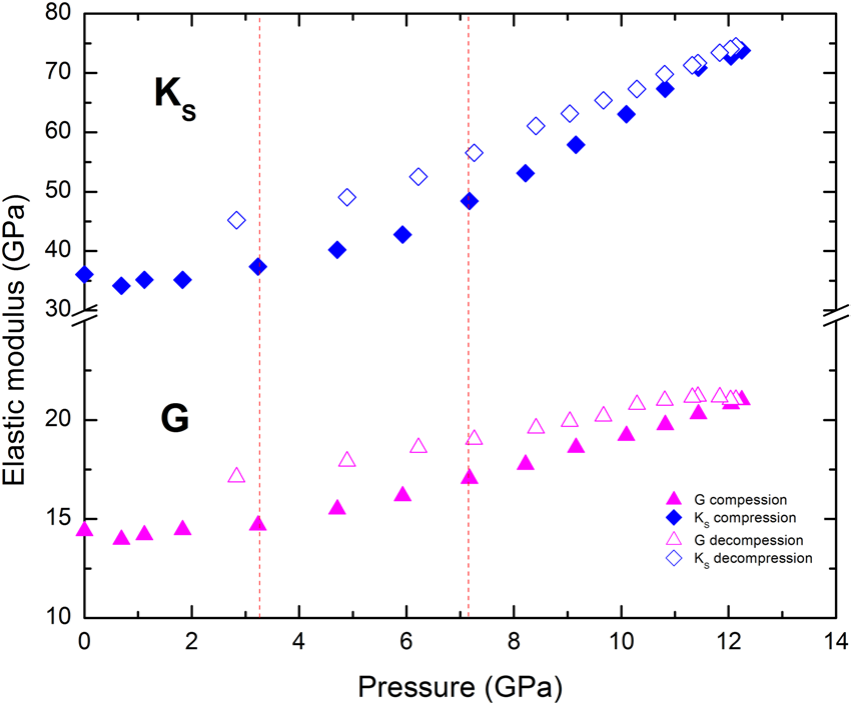
Bulk (blue diamonds) and shear (magenta triangles) moduli of the La_32_Ce_32_Al_16_Ni_5_Cu_15_ MG as a function of pressure at ambient temperature. Solid symbols are data on pressurization and open symbols are data on depressurization. Error bars are about the size of the symbols.

The calculated bulk (*K*_*S*_ = *ρ*(*ν*_*P*_^*2*^ *−4ν*_*S*_^*2*^ */3*) and shear (*G* = *ρν*_*S*_^*2*^) moduli at each pressure on compression and decompression are displayed in Fig. 3. The pressure derivatives of the elastic bulk and shear moduli were obtained by fitting the *P* and *S* wave velocities to the 3^rd^ order finite strain equations of state^34^ (see Method) in three pressure ranges of 0–3.2 GPa, 3.2–7.2 GPa and 7.2–12.3 GPa using the densities at 0, 3.2, and 7.2 GPa, respectively, as their corresponding initial values. At low pressures (0–3.2 GPa), a result of (∂*K*_*s*_/∂*P*) = 1.2(1) and (∂*G*/∂*P*) = 0.31(3) is obtained; in the intermediate pressure region (3.2–7.2 GPa), the pressure derivatives of the bulk and shear moduli increase to 2.6(2) and 0.63(3), respectively; and within 7.2–12.3 GPa, *K*_*s*_ and G increase with pressure at much higher rates of (∂*K*_*s*_/∂*P*) = 5.5(1) and (∂*G*/∂*P*) = 0.86(3). On decompression, both bulk and shear moduli show clear evidence of hysteresis and decrease monotonically with pressure. In comparison with the data obtained during compression, slope changes in the bulk and shear moduli on decompression are not obviously noticeable within experimental uncertainties. Thus, a single finite strain fit was used to analyze all the velocity data collected on decompression, yielding (∂*K*_*s*_/∂*P*) = 3.1(1) and (∂*G*/∂*P*) = 0.40(2).

## Discussion

In previous studies, the relations between volume and pressure for metallic glasses have been mostly limited to assessments from analyses of the position of the first sharp diffraction peak (FSDP), with the assumption that the first peak position in momentum transfer (*q*_*1*_), sampling primarily MRO, correlates with the specific volume of glass by power law relationship^20, 21^. For La_32_Ce_32_Al_16_Ni_5_Cu_15_, X-ray diffraction study has been conducted in a previous study and a FSDP-derived compression curve has been reported up to 40 GPa^9^. By comparison, the densities (specific volumes) from this study represent the true bulk density under pressure, and its high precision is ensured by the intrinsically precise travel time measurements of ultrasonic interferometry techniques.

**Figure 4:**
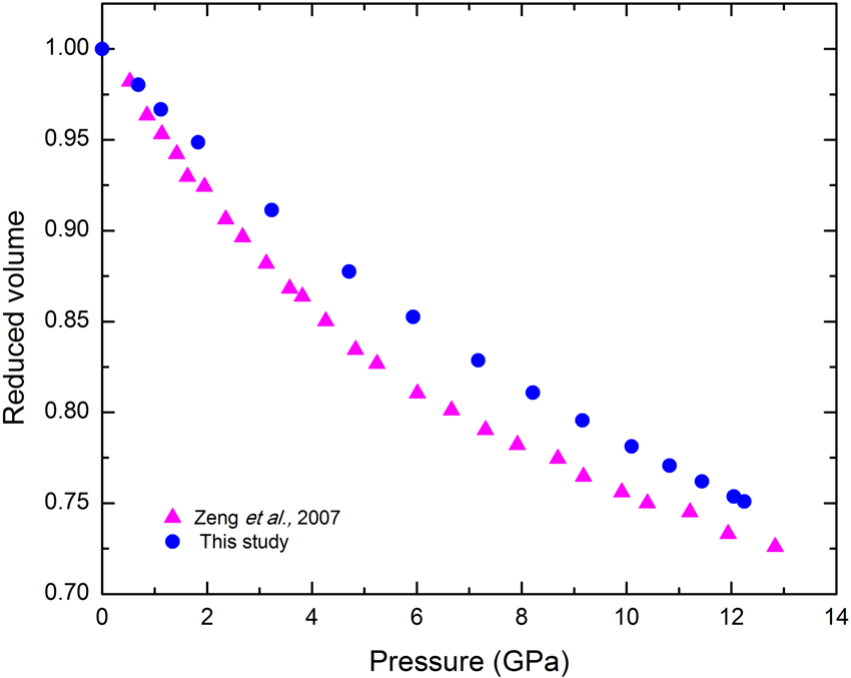
The specific volume changes as a function of pressure of Ce-based metallic glasses. For comparison, the specific volume derived from FSDP in ref. 7 is also indicated (magenta triangles).

We compare the P-V relation of La_32_Ce_32_Al_16_Ni_5_Cu_15_ from our measurements with that derived from FSDP reported in previous X-ray diffraction study in Fig. 4. Clearly, the estimation based on FSDP using V/V_0_ ~ (*q*_*0*_/*q*_*1*_)^3^ (subscript zero denotes zero pressure) yields a compression curve that increasingly deviates from our experimental data, reaching ~4% at ~12 GPa. The comparison indicates that (*q*_*0*_/*q*_*1*_)^3^ gives an overestimated specific volume change at all pressures, consistent with the nature of lacking long range order in amorphous materials. If the non-cubic power law with an exponent of 2.3–2.5 observed on Zr-based and other metallic glasses is applied^20, 21^, a better agreement with the current data can be achieved, but deviations are still noticeable. On the other hand, despite the ambiguities in the physical meaning of the exponent in the power law, a qualitatively consistent feature revealed by both FSDP and the current data is that the more rapid compression at low pressures (<5–7 GPa) may be suggestive of the occurrence of a non-first order LDA-HDA transition.

By comparison, the markedly different pressure derivatives of the bulk moduli in different pressure regimes in Fig. 3 clearly signifies that the Ce-based MG sample in this study undergoes a transition in compression mechanisms, which can be interpreted as a LDA state at low pressure (<GPa) and HDA state above 7 GPa, together with a mixed LDA and HDA state within 3–7 GPa. According to previous X-ray absorption studies^14^, the ratio of 4*f* ^*0*^ to 4*f* ^*1*^ components in Ce-based MG increases continuously from 1.7 to 3.2 GPa and reaches a plateau above 5 GPa, implying that a fully itinerant state has resulted in this pressure range. Note that the pressures where the gradual and continuous delocalization of 4*f* electrons is observed correlate well with the range where elastic wave velocities exhibit softening/weakening behavior. We thus conclude that a cohort rearrangement of constituent atoms during the LDA-HDA transition induced by the 4*f* delocalization is likely to be responsible for the anomalous behavior in the bulk elastic properties (Fig. 3).

In the literature, many different models have been proposed to describe the metallic glasses, ranging from Bernal’s dense random packing of hard spheres^35, 36^, the SRO/MRO solute-centered-clusters model consisting of solvent atoms in the first coordination shell and MRO of topological packing of SRO clusters packing schemes involving fcc, hcp, and icosahedral packing as in quasicrystals^17, 19, 37^, as well as a self-similar packing fractal network in the MRO^20, 22^. A more comprehensive review can be found elsewhere^30^. While it is impossible to examine all possible models with the current new data, an attempt to gain insights into the atomistic structure on the scale of SRO and MRO was only made following the solute-center cluster model.

According to Sheng’s model^17^, in the current La_32_Ce_32_Al_16_Ni_5_Cu_15_ metallic glass, Ce and La atoms summing up to 64% of the total atomic composition are the solvent atoms surrounding the solute atoms Al, Ni and Cu. The SRO consists of single solute atom Al-, Cu- and Ni-centered clusters; when the concentration of the solute species increases to beyond the maximum that a single-solute-centered cluster can contain, pairing of neighboring solute atoms and the formation of extended clusters become unavoidable. Thus, the high solute concentration (36%) in La_32_Ce_32_Al_16_Ni_5_Cu_15_ metallic glass may lead to a network-like arrangement of the solute atoms (see Ni_63_Nb_37_ model in ref. 11), with the solute-centered clusters and extended clusters being considered as rigid units while the extra La and Ce atoms outside the clusters are “free” or “glue” atoms between clusters. Under applied pressure, while 4*f* electrons of Ce undergo delocalization as observed in X-ray absorption studies^12, 14^, it occurs more gradually and spreads over a wider pressure range than that observed in the elemental metal, presumably due to the heterogeneous local stress environment in the multicomponent metallic glass. The “free volume” generated by the volume change associated with the electronic transition of Ce causes the La-La, La-Ce or Ce-Ce bond lengths to shorten and enables the solute-centered clusters to facilitate mobility and distortion. Effectively, the structure can evolve in a similar fashion to what occurs in network glasses, such as SiO_2_, in which the densification under pressure proceeds with decreased Si-O-Si angles and the tetrahedral ring sizes to achieve an increased coordination number in Si^29^. For GeSe_2_ glass, the minimum in *S* wave velocity is observed at 4 GPa. The network is floppy due to the breakup of cross-linking elements^23^. Based on the model above, the velocities variations in ν_P_ and ν_S_ can be interpreted as resulting from topological rearrangements of network-like clusters (MRO) on compression, analogous to the softening mechanism observed in network glasses. At pressures above 5 GPa when the 4*f* ^*0*^ and 4*f* ^*1*^ ratio in Ce reaches a plateau, the clusters at SRO/MRO behave much like rigid units and further increasing pressure is mainly accommodated by the shrinkage of the clusters size due to bond length change, leading to a more homogeneous compression like isotropic crystalline solids. Upon decompression, the elastic moduli decrease smoothly without discontinuities, indicating that the topological arrangements of clusters are not reversible. This irreversible MRO modification of clusters results in residual densification of the La_32_Ce_32_Al_16_Ni_5_Cu_15_ metallic glass.

## Methods

The La_32_Ce_32_Al_16_Ni_5_Cu_15_ metallic glass rod was prepared using copper mold casting method as described in ref. 38. Ingots were prepared by arc-melting a mixture of pure La (99.5 at.%), Ce (99.5 at.%), Al (99.95 at.%), Ni (99.98 at.%) and Cu (99.9 at.%) in a Zr-gettered argon atmosphere and the ingot was remelted five times to make the composition homogenous. The amorphous structure of the sample was examined by X-ray diffraction (XRD) on the transverse section of sample rods using a Thermo ARL X’Tra diffractometer with CuK_α_ radiation at 45 kV. A sample disk of 0.504(1) mm in length and 2.440(1) mm in diameter was cut from the rod, and both faces of the sample were polished flat and parallel. The density of the sample, 6.303 g cm^−3^, was measured using the Archimedes’ method with an accuracy of about 0.005 g cm^−3^. Ultrasonic measurements were performed in a 1000-ton uniaxial split cylinder apparatus (USCA-1000) with a Walker type cylindrical multi-anvil module up to 12.3 GPa at room temperature. Details of the experimental set-up for the ultrasonic interferometry can be found elsewhere^39^. The sample that embedded in the center of the MgO octahedral cell was surrounded by a lead sleeve. Disks of Teflon and lead were inserted to provide a pseudo-hydrostatic pressure environment and protect the sample from cracking at high pressures. On the other end, an alumina rod (3.2 mm in diameter and 3.8 mm in length) served as an acoustic buffer rod and an *in-situ* pressure marker (see Supplementary Fig. S1)^40^. A dual-mode LiNbO_3_ transducer (50 MHz resonant frequency for *P* wave and 30 MHz for *S* wave) was mounted on a tungsten carbide cube (edge length 25.4 mm) with one corner truncated into a triangular surface (edge length 8 mm) for generating and receiving compressional (*P*) and shear (*S*) wave signals simultaneously. A thin gold foil (2 μm thickness) was placed between the buffer rod and the sample, as well as between the buffer rod and tungsten carbide anvil to enhance mechanical bonding and to optimize the acoustic energy propagation into the sample^41^. Two-way travel times were determined by the pulse echo overlap (PEO) methods. Details about transfer function method and its advantage of processing data have been discussed elsewhere^42^. Raw travel times were corrected for the effect of the gold bond and the magnitude of this correction is approximately 0.5 ns for *P* waves and 0.13 ns for *S* waves. The elastic wave velocities were calculated from these travel times and the changes of sample length with pressure were accounted for by using Cook’s methods^43^:

1 $$\frac{\,{L}_{0}}{L}=1+\frac{1+\alpha \gamma T}{3{\rho }_{0}{{L}_{0}}^{2}}\int \frac{1}{\frac{1}{{({t}_{p})}^{2}}-\frac{4}{{(3{t}_{s})}^{2}}}dP$$

where *ρ*_0_ is the density, *L*_0_ and *L* are sample length at ambient conditions and high pressure, respectively, *t*_*p*_ and *t*_*s*_ are one way travel times of *P* and *S* waves through the sample, *α* is the thermal expansion coefficient and *γ* is the Grüneisen parameter. (1+*αγT*) = *Cp/Cv* is the ratio of specific heats at constant pressure and volume, which is around 1.01 for most materials^32^. All the experimental data can be found as Supplementary Tables S1 and S2.

## Electronic supplementary material

Pressure Calibration and experimental data
