# Supplementary material for: Elastic Anomaly and Polyamorphic Transition in (La, Ce)-based Bulk Metallic Glass under Pressure
Source: Sci Rep. 2017 Apr 7;7:724. doi: 10.1038/s41598-017-00737-0 (PMC5429654; doi:10.1038/s41598-017-00737-0)
Supplement: Supplementary file 1 — Pressure Calibration and experimental data [file 41598_2017_737_MOESM1_ESM.pdf]

Supplementary Information

Elastic Anomaly and Polyamorphic Transition in (La, Ce)-based Bulk Metallic Glass under Pressure

Xintong Qi, [1,*] Yongtao Zou,[2] Xuebing Wang,[1] Ting Chen,[1] David O. Welch,[3] Jianzhong Jiang,[4] Baosheng Li[5]

[1] Department of Geosciences, Stony Brook University, Stony Brook, N.Y., 11794, United States

[2] State Key Laboratory of Superhard Materials, College of Physics, Jilin University, Changchun, 130012, P. R. China

[3]Condensed Matter Physics and Materials Science Department, Brookhaven National Laboratory, Upton, N.Y. 11973, United States

[4] International Center for New-Structured Materials (ICNSM), Laboratory of New-Structured Materials, State Key laboratory of Silicon Materials, and School of Materials Science and Engineering, Zhejiang University, Hangzhou, 310027, P.R. China

[5]Mineral Physics Institute, Stony Brook University, Stony Brook, N.Y. 11794, United States

* Corresponding author: Xintong Qi (xintong.qi@stonybrook.edu)

S1. Pressure Calibration

The cell pressure of the USCA-1000 multi-anvil apparatus was calibrated using the S wave travel times of Al_2O_3 buffer rod. The relationship of S wave travel times as a function of pressure can be expressed in the equation:

$$P = 1.5015 \times 10^4 \times (\frac{T_S}{T_{S0}})^3 - 4.3480 \times 10^4 \times \left(\frac{T_S}{T_{S0}}\right)^2 + 4.1718 \times 10^4 \times \left(\frac{T_S}{TS0}\right) - 1.3251 \times 10^4 \quad \text{(S1)}$$

where T is the measured S wave travel time of alumina at high pressure and substript"0" denotes zero pressure.

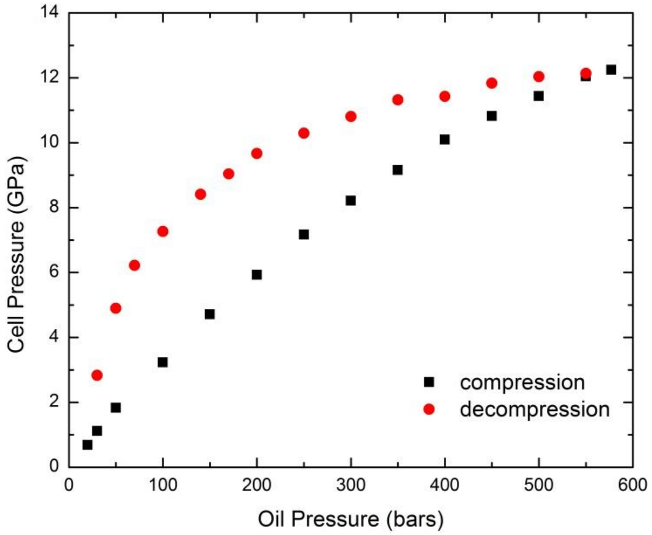

Figure S1. Al_2O_3 buffer rod travel time pressure calibration.

Supplementary Table S1. Experimental results for of the $La_{32}Ce_{32}Al_{16}Ni_5Cu_{15}$ during compression. The uncertainties in velocities and elastic moduli are approximately 0.5 %.

Pressure (GPa)	$2t_P$ (μs)	$2t_S$ (μs)	length (mm)	V_P (km/s)	V_S (km/s)	G (GPa)	K_S (GPa)	ρ (g/cm^3)
0.7	0.3495(2)	0.6794(2)	0.501(1)	2.87	1.47	14.0	34.1	6.43(1)
1.1	0.3460(2)	0.6754(2)	0.498(1)	2.88	1.48	14.2	35.2	6.52(1)
1.8	0.3460(2)	0.6714(2)	0.495(1)	2.86	1.48	14.5	35.2	6.64(1)
3.2	0.3405(2)	0.6704(2)	0.489(1)	2.87	1.46	14.7	37.4	6.91(1)
4.7	0.3315(2)	0.6569(2)	0.483(1)	2.91	1.47	15.5	40.2	7.18(1)
5.9	0.3240(2)	0.6464(2)	0.478(1)	2.95	1.48	16.2	42.8	7.39(1)
7.2	0.3095(2)	0.6324(2)	0.473(1)	3.06	1.50	17.0	48.4	7.60(1)
8.2	0.2990(2)	0.6219(2)	0.470(1)	3.14	1.51	17.8	53.1	7.77(1)
9.2	0.2890(2)	0.6094(2)	0.467(1)	3.23	1.53	18.6	57.9	7.92(1)
10.1	0.2800(2)	0.6014(2)	0.464(1)	3.32	1.54	19.2	63.0	8.06(1)
10.8	0.2730(2)	0.5944(2)	0.462(1)	3.39	1.55	19.8	67.3	8.18(1)
11.4	0.2675(2)	0.5874(2)	0.460(1)	3.44	1.57	20.3	70.9	8.27(1)
12.0	0.2645(2)	0.5814(2)	0.459(1)	3.47	1.58	20.8	72.8	8.36(1)
12.2	0.2630(2)	0.5789(2)	0.458(1)	3.48	1.58	21.0	73.8	8.39(1)

Supplementary Table S2. Experimental results for of the $La_{32}Ce_{32}Al_{16}Ni_5Cu_{15}$ during decompression. The uncertainties in velocities and elastic moduli are approximately 0.5 %.

Pressure (GPa)	$2t_P$ (μs)	$2t_S$ (μs)	length (mm)	V_P (km/s)	V_S (km/s)	G (GPa)	K_S (GPa)	ρ (g/cm^3)
12.1	0.2620(2)	0.5784(2)	0.458(1)	3.50	1.58	21.0	74.5	8.38(1)
12.0	0.2625(2)	0.5784(2)	0.459(1)	3.49	1.59	21.0	74.1	8.37(1)
11.8	0.2630(2)	0.5764(2)	0.459(1)	3.49	1.59	21.2	73.4	8.34(1)
11.4	0.2650(2)	0.5754(2)	0.460(1)	3.47	1.60	21.2	71.7	8.30(1)
11.3	0.2655(2)	0.5759(2)	0.460(1)	3.47	1.60	21.1	71.3	8.28(1)
10.8	0.2675(2)	0.5774(2)	0.461(1)	3.45	1.60	21.0	69.8	8.22(1)
10.3	0.2710(2)	0.5794(2)	0.462(1)	3.41	1.60	20.8	67.3	8.16(1)
9.7	0.2745(2)	0.5869(2)	0.464(1)	3.38	1.58	20.2	65.4	8.08(1)
9.0	0.2780(2)	0.5899(2)	0.465(1)	3.35	1.58	19.9	63.2	8.01(1)
8.4	0.2815(2)	0.5939(2)	0.467(1)	3.32	1.57	19.6	61.1	7.92(1)
7.3	0.2895(2)	0.6009(2)	0.470(1)	3.25	1.56	19.0	56.6	7.77(1)
6.2	0.2970(2)	0.6054(2)	0.473(1)	3.18	1.56	18.6	52.5	7.63(1)
4.9	0.3045(2)	0.6144(2)	0.477(1)	3.13	1.55	17.9	49.1	7.43(1)
2.8	0.3130(2)	0.6239(2)	0.484(1)	3.09	1.55	17.1	45.2	7.11(1)
